# Supplementary figures and images for: Impact of mini-driver genes in the prognosis and tumor features of colorectal cancer samples: a novel perspective to support current biomarkers
Source: PeerJ. 2023 May 16;11:e15410. doi: 10.7717/peerj.15410 (PMC10198153; doi:10.7717/peerj.15410)

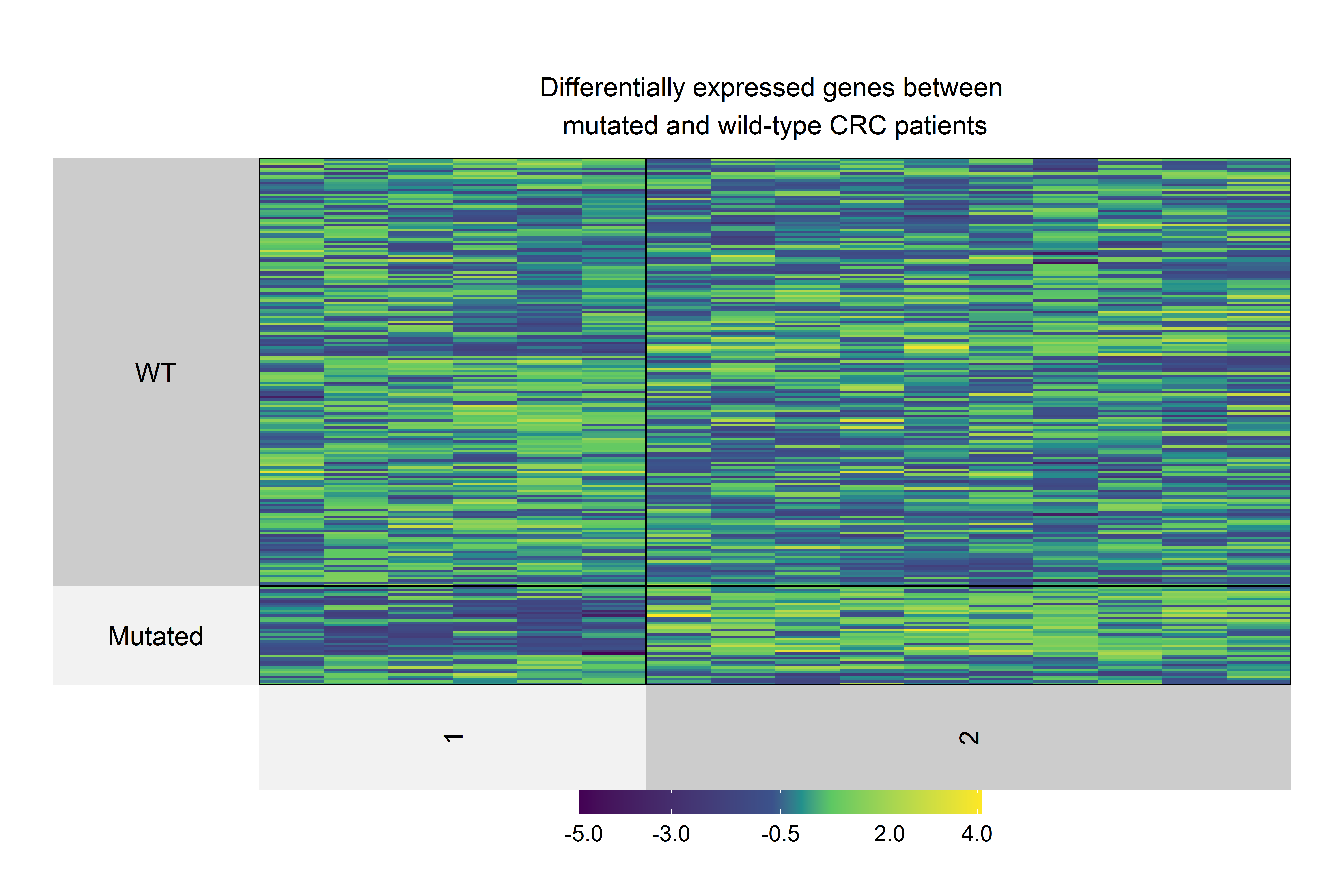

Supplement: Supplemental Information 1 — Differentially expressed genes were clustered in two groups: Upregulated and Downregulated. Upregulated genes were DOCK3, FN1, ADAMTS2, AHNAK, AHNAK2, DNAH7, NBEA, SACS, SMAD4, and VWF genes, whereas AMER1, DIDO1, LRP1, LRP1B, RNF43, and TG were downregulated. [file peerj-11-15410-s001.png]
